# Evaluation of right and left ventricular function using speckle tracking echocardiography in patients with arrhythmogenic right ventricular cardiomyopathy and their first degree relatives

**DOI:** 10.1186/1476-7120-10-37

**Published:** 2012-09-19

**Authors:** Meriam Åström Aneq, Jan Engvall, Lars Brudin, Eva Nylander

**Affiliations:** 1 Division of Clinical Physiology, Department of Medical and Health Sciences, Faculty of Health Sciences, Department of Clinical Physiology, County Council of Östergötland, Linköping University, Linköping, Sweden; 2 Center for Medical Image Science and Visualization, Linköping University, Linköping, SE-58185, Sweden; 3 Department of Clinical Physiology, Kalmar county hospital, Kalmar, Sweden; 4 Department of Clinical physiology, Heart centre, University hospital, Linköping, 581 85, Sweden

**Keywords:** Arrhythmogenic right ventricular cardiomyopathy, Right ventricle, Strain, Echocardiography, Right ventricular function

## Abstract

**Introduction and aim:**

The identification of right ventricular abnormalities in patients with arrhythmogenic right ventricular cardiomyopathy (ARVC) in early stages is still difficult. The aim of this study was to investigate if longitudinal strain based on speckle tracking can detect subtle right (RV) or left ventricular (LV) dysfunction as an early sign of ARVC.

**Methods and results:**

Seventeen male patients, fulfilling Task force criteria for ARVC, 49 (32–70) years old, nineteen male first degree relatives 29 (19–73) y.o. and twenty-two healthy male volunteers 36 (24–66) y.o participated in the study. Twelve-lead and signal-averaged electrocardiograms were recorded. All subjects underwent echocardiography. LV and RV diameters, peak systolic velocity from tissue Doppler and longitudinal strain based on speckle tracking were measured from the basal and mid segments in both ventricles. RV longitudinal strain measurement was successful in first degree relatives and controls (95 resp. 86%) but less feasible in patients (59%). Results were not systematically different between first degree relatives and controls. Using discriminant analysis, we then developed an index based on echocardiographic parameters. All normal controls had an index < l while patients with abnormal ventricles had an index between 1–4. Some of the first degree relatives deviated from the normal pattern.

**Conclusion:**

Longitudinal strain of LV and RV segments was significantly lower in patients than in relatives and controls. An index was developed incorporating dimensional and functional echocardiographic parameters. In combination with genetic testing this index might help to detect early phenotype expression in mutation carriers.

## Introduction

Arrhythmogenic right ventricular cardiomyopathy (ARVC) is characterized by fibro-fatty substitution of the myocardium in the right (RV) and, not infrequently, in the left ventricle (LV). The loss of normal myocardium and the development of scar is associated with electrical instability manifested as ventricular arrhythmia and potential sudden death [1]. A less common manifestation is right ventricular dysfunction causing heart failure and thromboembolism. ARVC is typically transmitted as an autosomal dominant trait with variable expressivity and penetrance [2]. Current guidelines recommend that all first degree relatives of a patient with ARVC undergo screening as sudden cardiac death may be the initial manifestation of the disease. During the past decade, several genes and loci have been identified as responsible for ARVC [3]. The availability of molecular genetic testing allows the identification of gene-positive individuals. However, because of the lack of knowledge about long-term outcome in genetically-affected relatives, lifelong follow-up of these relatives is required for early detection of signs of disease.

In addition to resting ECG, imaging of the RV by two-dimensional echocardiography (2D) is the most important screening method. The identification of RV abnormalities using echocardiography is still a major challenge because of the geometric shape of the RV and the patchy involvement of the right ventricular wall with subtle abnormalities at an early stage.

Recently, a 2D strain echocardiographic method [4-7] has been introduced that measures myocardial deformation by tracking localized acoustic markers frame by frame (speckle tracking). This method has been used for noninvasive assessment of regional myocardial strain in the left [8-10] and right [11] ventricle, avoiding the angular sensitivity of tissue Doppler echocardiography.

We hypothesized that right and left ventricular longitudinal strain derived from speckle tracking could be used as a sensitive tool for the detection of subclinical ventricular dysfunction in first degree relatives of ARVC patients.

The aim of this study was (1) to test the feasibility of the method when applied on the RV in patients with ARVC and their relatives, (2) to investigate whether first degree relatives of ARVC patients have reduced longitudinal strain by speckle tracking as an early sign of myocardial dysfunction, compared to controls.

## Material and methods

### Study population

ARVC affects females less often than males [12]. In this study we have focused upon male patients as well as their male relatives and matched controls, to avoid potential gender differences in myocardial functional parameters.

#### ARVC patients

Patients were referred from hospitals and primary care centres in the southeast of Sweden for investigation and follow-up of ARVC. Since 1994, 19 males have been diagnosed with ARVC based on fulfilment of the Task Force Criteria of the European Society of Cardiology [13]. Two patients underwent heart transplantation before start of this study and were excluded. The remaining 17 males were included in the study.

#### First degree relatives

All first degree male relatives of ARVC patients (children, parents or siblings), 18 years or older, underwent screening at our tertiary care referral centre. One of them fulfilled criteria for ARVC at the screening visit and was excluded, resulting in 19 relatives, not fulfilling original Task Force criteria, in the study. Kinship has been established from voluntary information from the patients and was not checked with DNA testing.

#### Controls

Twenty-four healthy male volunteers were recruited. All were asymptomatic and lacked family history of premature cardiovascular disease. No one was on cardiac medication. The selection of controls was based on matching age to that of the group of relatives, aiming at an age difference < 5 years. Two intended controls were excluded, one due to left bundle branch block (LBBB) on ECG and one due to an abnormal ST-reaction on exercise testing leaving 22 for inclusion. Ethical approval for the study was obtained from the Regional Ethical Review Board in Linköping.

### Procedure

The three separate groups - ARVC patients, their first degree relatives and healthy controls - were evaluated by medical history, blood pressure measurement and twelve-lead electrocardiography (ECG). A bicycle exercise test was performed for ruling out significant heart disease. A signal-averaged ECG (SAECG) using 40 Hz high-pass filter was recorded in patients and relatives but not in the healthy volunteers. Positive late potentials were diagnosed when at least two of the three criteria proposed by Breithardt [14] were present.

Echocardiography was performed with the subjects at rest in the left lateral decubitus position using Vivid 5 or Vivid 7 echocardiographic scanners (GE Medical Systems, Horten, Norway). All images were stored digitally and analyzed on the Echo Pac work station (GE Medical Systems, EchopacPC, version 6.0.1).

A comprehensive 2D, colour, pulsed, and continuous-wave Doppler examination was performed following the recommendations of the American Society of Echocardiography for transthoracic studies. Parasternal long-axis views were used to derive M-mode measurement of left ventricular (LV) end-diastolic dimension, and to determine aortic valve opening (AVO) and closure (AVC). All timing information was in relation to the ECG. The right ventricular (RV) inflow and outflow diameter was measured from the 2D images and indexed to body surface area (RVIT/BSA and RVOT/BSA). Tricuspid annular motion (TAPSE) was recorded at the right ventricular free wall using cross sectional guided M-mode.

Colour tissue Doppler myocardial imaging of the LV and RV was performed in the apical four-chamber view, at high frame rate (>100 frames/s). Three consecutive beats were recorded for off line analysis. Mitral and tricuspid annular systolic velocities (Sw) as well as early (Ew) and late diastolic velocities (Aw) were measured in the lateral LV wall, septum and RV free wall with a region of interest (ROI) size of 3 mm.

#### 2D-strain echocardiography

**Figure 1:**
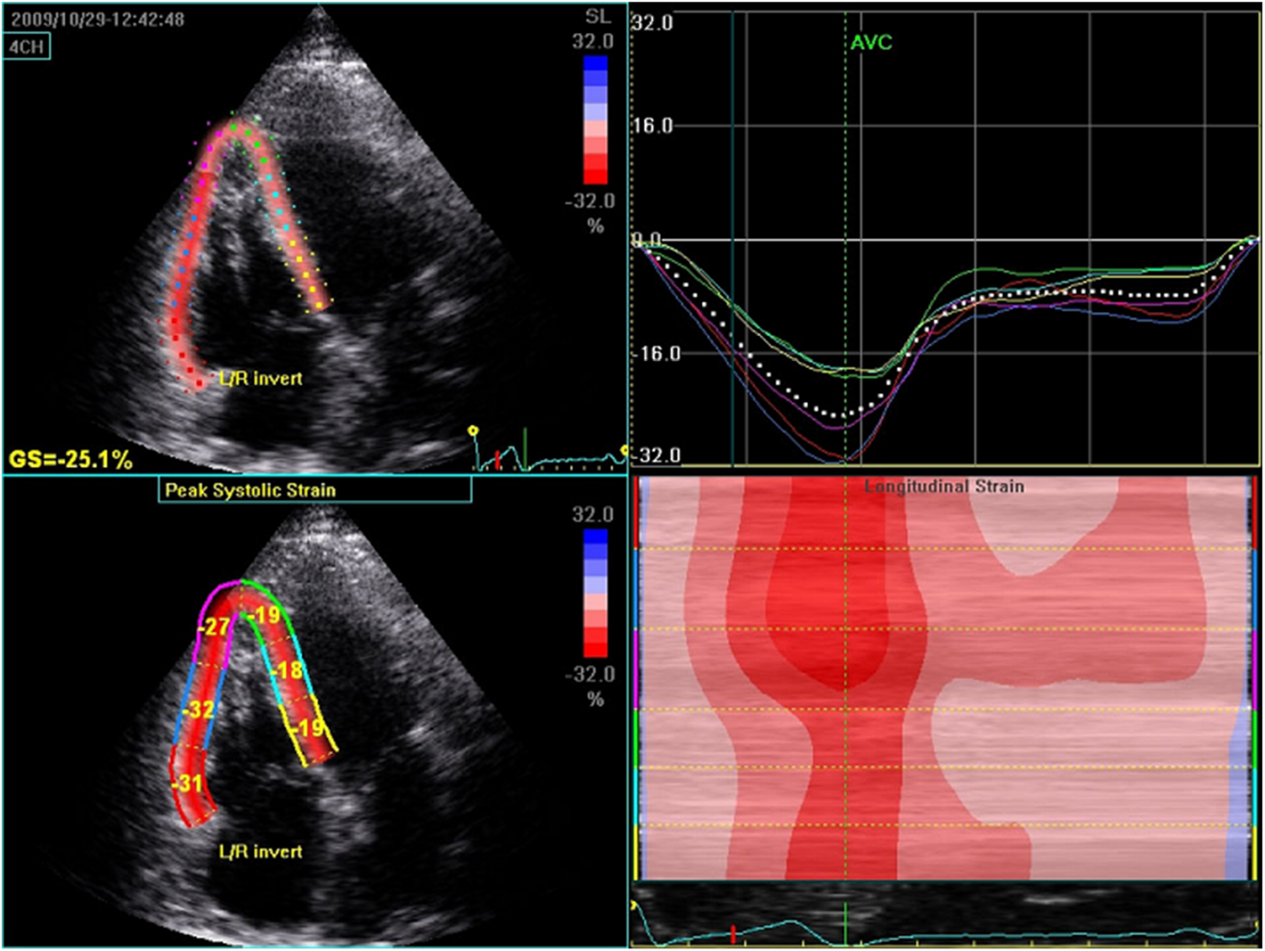
**Determination of longitudinal strain in an apical four chamber view.** Left side: Colour display of peak systolic strain. The right side shows average segmental strain graphically displayed (upper) and an M-mode representation of peak systolic strain (lower).

The two dimensional four chamber view was recorded with a frame rate ranging between 40 and 70 frames/s. The endocardial border was traced offline and the ROI was manually adjusted to include the myocardial wall. The software then automatically tracked wall motion over the entire cardiac cycle and measurements were accepted from segments of good tracking quality. The observer was allowed to manually readjust some but not all aspects of the tracking procedure. Systolic longitudinal strain was assessed from the basal and mid segments in the lateral LV wall, septum and RV free wall, Figure 1.

All strain measurements were repeated by the same observer who was blinded to the initial values, at least 5 weeks after the first analysis, in a random sample of 12 echocardiographic studies (4 from each patient group). To define interobserver variability, measurements in the same random sample were assessed by a second independent observer blinded to the results obtained by the previous investigator. The investigator was blinded for the analysis of echocardiographic data.

### Data analysis and statistics

Results were expressed as median (range) for patient details (Table 1) and mean and standard deviation for echocardiographic variables, which were reasonably well normally distributed.

**Table 1:** Subject clinical details

	**ARVC (n = 17)**	**Relatives (n = 19)**	**Controls (n = 22)**	**P-value**	P-value*
	**Median (range)**	**Median (range)**	**Median (range)**		
**Age (years)**	49 (32–70)	29 (19–73)	36 (24–66)	0.024	0.445
**Heart rate (bpm)**	58 (45–90)	62 (49–88)	68 (45–94)	0.178	0.121
**BMI (kg/m**^**2**^**)**	25.3 (20.2–32.2)	24.3 (20.5–42.6)	22.5 (18.8–28.1)	0.177	0.371
**BSA (m**^**2**^**)**	1.96 (1.65–2.39)	2.00 (1.73–2.64)	1.97 (1.71–2.25)	0.898	0.990
**SBP (mmHg)**	130 (100–170)	130 (110–155)	120 (105–140)	0.078	0.045
**DBP (mmHg)**	80 (60–95)	80 (60–90)	70 (60–90)	0.002	0.018
**Arrhythmia-related symptoms**	13 (76%)	2 (11%)	0	-	-

Differences between groups analyzed using non-parametric ANOVA.

* Controls vs. relatives using Mann–Whitney *U*-test.

BMI is body mass index, BSA is body surface area, SBP and DBP are systolic respective diastolic blood pressure.

Inter- and intraobserver variability was expressed as standard error of a single determination (S_method_) using the formula first proposed by Dahlberg [15]. S_method_ was also expressed as % over all means. The coefficient of variation (COV) is the standard deviation of the differences divided by the mean of the measurements and expressed as a percentage.

## Results

### Clinical characteristics and ECG

Two of the 19 relatives (11%) had a history of occasional palpitations but ventricular ectopy was not seen on the resting ECG or at the exercise test.

The resting ECG did not demonstrate inverted T-waves beyond precordial lead V1 in the relatives or in the control group while abnormal, negative T-waves in right sided precordial leads were seen in 13 patients with ARVC (76%). In addition, two had right bundle branch block. One person among the relatives, 3 among controls and 4 patients had incomplete RBBB. Late potentials (defined as fulfilment of at least 2 of 3 criteria) were found in 9/14 (64%) of ARVC patients, but in 2 patients SAECG was not recorded because of frequents arrhythmias and in one case due to ongoing cardiac pacing. Among the first degree relatives 3/19 (16%) had positive late potentials. No one in the control group had positive late potentials.

Three (16%) of the first degree relatives were on beta-blockers for mild arterial hypertension. In the ARVC group 13 (70%) had medication for ventricular arrhythmia, arterial hypertension or heart failure. Nine patients (53%) had an implantable cardioverter defibrillator (ICD).

### Echocardiography

Longitudinal strain measurement using speckle tracking was successful in the left ventricular walls in 15 of 17 (88%) patients, 18 of 19 (95%) first degree relatives and 19 of 22 (86%) controls. For the right ventricular free wall, 2D-strain measurement was feasible in both segments in 10 of 17 (59%) patients. However, two patients had unreliable values in the mid RV). In first degree relatives and controls, 2D-strain in the RV free wall was obtained in 18 of 19 (95%) and 19 of 22 (86%), respectively. Missing data was mostly due to signal dropout, artifact or a very thin wall in the region of interest making wall motion tracking unreliable*.*

**Figure 2:**
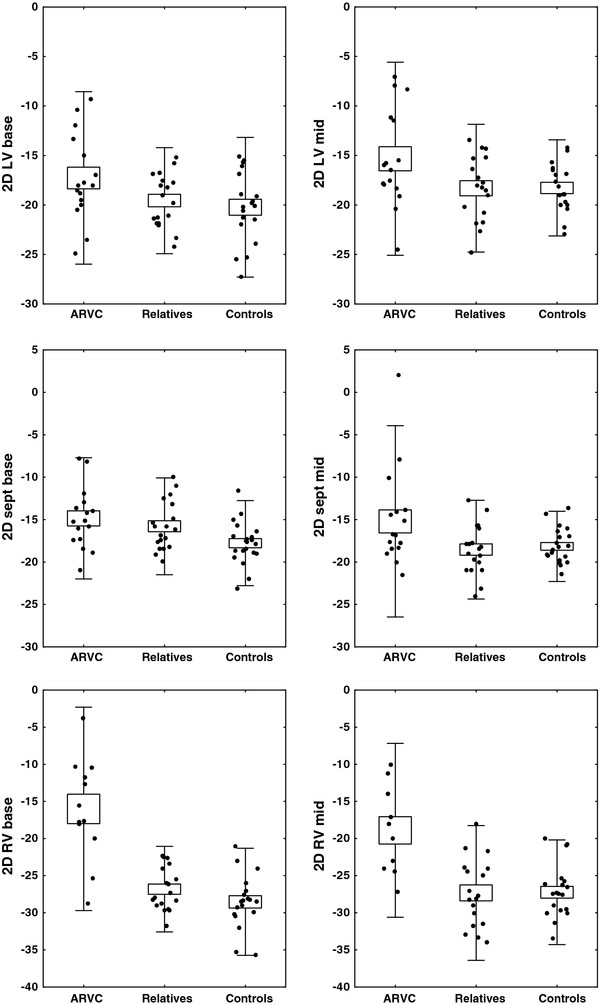
**Longitudinal strain using speckle tracking in the base and mid lateral left ventricular (LV), septum (sept) and right ventricular free (RV) wall in patients with ARVC, their first degree relatives and controls.** Black dots represent subjects. Boxes, mean values and standard deviation.

The left ventricular diameter was significantly larger in the relatives compared to the controls and the patients. A significantly lower myocardial velocity was detected in the left lateral wall compared to the septum, lowest in the patients and highest in the controls. Except for a lower longitudinal strain value in the basal septum in relatives compared to controls, there were no significant differences between the first degree relatives and controls for the remaining parameters (Table 2 and Figure 2).

**Table 2:** Echocardiographic findings in the three groups

	**ARVC (n = 17)**	**Relatives (n = 19)**	**Controls (n = 22)**	P-value*
	**Mean (SD)**	**Mean (SD)**	**Mean (SD)**	
**LV diameter (mm)**	52.6 (6.4)	54.6 (4.4)	51.3 (2.9)	**0.009**
**RVOT/BSA (cm/m**^**2**^**)**	2.0 (0.4)	1.6 (0.2)	1.6 (0.2)	0.981
**RVIT/BSA (cm/m**^**2**^**)**	2.3 (0.5)	1.9 (0.2)	1.8 (0.2)	0.674
**LVOT VTI (cm)**	18.8 (5.5)	21.3 (2.5)	20.9 (2.4)	0.555
**TAPSE (mm)**	19.1 (5.0)	25.3 (2.9)	25.3 (3.0)	0.963
**Sw lat. LV (cm/s)**	6.6 (2.3)	7.3 (1.2)	8.5 (1.9)	**0.018**
**Sw Sept. (cm/s)**	5.7 (1.6)	6.6 (1.0)	7.1 (0.9)	0.115
**Sw RV free wall (cm/s)**	8.2 (2.5)	11.2 (1.9)	11.6 (1.7)	0.545
**E/A LV**	1.3 (0.5)	1.8 (0.6)	1.7 (0.4)	0.600
**E/E’ LV**	8.8 (3.2)	6.4 (2.1)	6.1 (1.6)	0.608
**2D strain LV base (%)**	−17.3 (4.4)	−19.6 (2.7)	−20.2 (3.5)	0.518
**2D strain LV mid (%)**	−15.3 (4.9)	−18.3 (3.2)	−18.3 (2.4)	0.968
**2D strain sept base (%)**	−14.9 (3.6)	−15.8 (2.9)	−17.8 (2.5)	**0.025**
**2D strain sept mid (%)**	−15.2 (5.6)	−18.5 (2.9)	−18.2 (2.1)	0.636
**2D strain RV base (%)**	−16.0 (6.8)	−26.8 (2.9)	−28.5 (3.6)	0.121
**2D strain RV mid (%)**	−18.9 (5.9)	−27.3 (4.5)	−27.2 (3.5)	0.945

* Controls vs. relatives using Student’s *t*-test.

Right ventricular outflow and inflow diameter related to BSA(RVOT/BSA resp.RVIT/BSA), Left ventricular outflow velocity time integral (LVOT VTI), tricuspid annular plane systolic excursion (TAPSE), systolic velocity at AV-plane in lateral LV (SwLV), septum (Sw sept) and RV wall (Sw RV).

The intraindividual methodological error was for strain 1.6 percentage points (PP) (COV = 8.2%), the same in various locations of the heart. The inter-individual error was, as expected, greater, 2.5 PP (COV = 12.7%), with 2D strain error in the mid level of the RV being significantly greater expressed in absolute but not in relative terms (3.94 PP, COV = 15.7%), otherwise no significant differences were found between different parts of the heart.

Based on measurement results from the patient group and the volunteers, a multivariate discriminant analysis was performed to detect differences between ARVC-patients and controls in order to investigate the relative role of different parameters from the right and left ventricle for the discrimination of the two groups. The dimensional and functional parameters used for the right ventricle were RVOT/BSA, RVIT/BSA, TAPSE, Sw and longitudinal strain based on speckle tracking of the basal and mid RV free wall segments. For the left ventricle the parameters were LV diameter, LVOT VTI, Sw from the lateral and septal walls, strain based on speckle tracking of the basal and mid segments of the septum and lateral wall. Significant components were reassembled to create separate indices for the right and left ventricles and recalculated for each individual.

The following discriminant function was obtained using parameters from the right ventricle:

$$\begin{array}{l} \text{Proposed}\;\text{index}\;\text{for}\;\text{the}\;\text{right}\;\text{ventricle}\\ =\text{ln(RVIT/BSA)*-Sw RV free wall}\\ \;\text{*}0.71\text{+2D strain RV base}\\ \;\text{*}0.852-2\text{D strain RV mid*}0.418\text{ -Agecat}\\ \;\text{*}1.276+12\text{.} \end{array}$$

The different age categories (Agecat) were defined as follows: 1 is ≤27, 2 is 28–38, 3 is 39–58 and 4 is >58 years of age and ln is the natural logarithm.

The corresponding equation for the left ventricle was:

$$\begin{array}{l} \text{Proposed}\;\text{index}\;\text{for}\;\text{the}\;\text{left}\;\text{ventricle}\;\\ \text{= LV diameter per BSA*}0.393\text{ - Sw lat LV}\\ \;\text{*}0.494\text{ - Sw sept*}0.011\text{ + }2\text{D strain sept base}\\ \;\text{*}0.261\text{ - Agecat*}0.028-3\text{.} \end{array}$$

These indices were applied to the group of first degree relatives.

**Figure 3:**
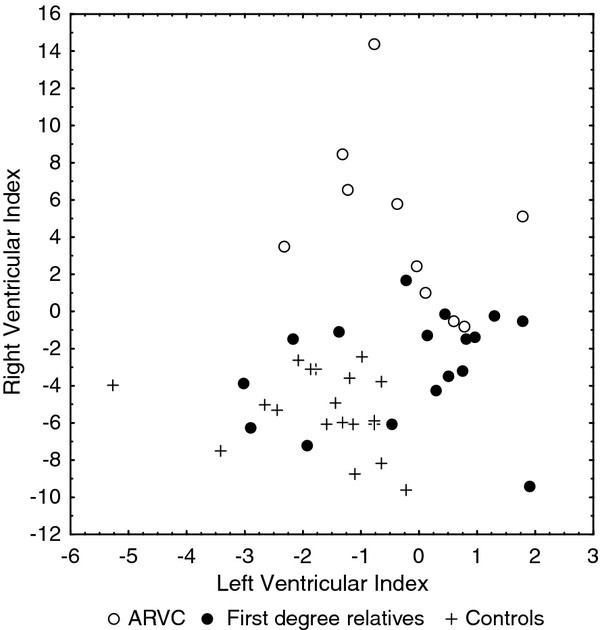
The distribution of measurements from first degree relatives in relation to ARVC patients and controls using a left and a right ventricular index calculated by discriminant analysis.

Results of the ARVC discrimination index are depicted in Figure 3. All individuals were also ranked according to their index for the right ventricle in order to identify relatives who had an echocardiographic pattern similar to that of the patients.

## Discussion

New methods for the assessment of myocardial function have broadened the utility of echocardiography in detecting and understanding the effect of contraction abnormalities of the heart, sometimes in subclinical phases of disease [16-18].

In this investigation we have studied RV and LV function using the speckle tracking method.

Speckle tracking was feasible in regions of interest from both ventricles in patients, relatives and controls. However, longitudinal strain measurements in the right ventricular free wall were more often unreliable in patients (41%) than in relatives and controls. This can be explained by the main pathologic feature of the disease, with myocardial degeneration causing thinning of the right ventricular wall, prohibiting tracking with the present version of the software. Using the same software, Teske et al. showed a higher feasibility [19,20] of speckle tracking measurements in their group of ARVC patients compared to ours. This could partly be explained by differences between groups in the severity of disease.

The inter- and intraobserver variability of speckle tracking measurements was low in both ventricles with low bias and low limits of agreement. As expected the interobserver variability was slightly higher than the intraobserver variability and both measurements were higher for the right ventricular free wall than for the septum and left ventricular lateral wall.

The current study shows that longitudinal speckle derived strain is reduced in the right as well as in the left ventricular walls in patients, confirming the biventricular involvement in ARVC [21-24]**,** but there was no systematic difference in-between relatives and controls.

Echocardiographic findings can be discrete in the early stages of the disease, complicating early diagnosis as Corrado et al. have pointed out [25].

ARVC is a progressive disease that can have variable phenotypic expression. Sometimes, malignant ventricular arrhythmia or sudden cardiac death are the initial manifestations.

On the cellular level, it is a disease of the desmosome which affects the cell-adhesion proteins leading to cardiomyocyte detachment and death. ARVC is an autosomal dominant disease in at least 50% of cases [13,26]. The recent development of genetic tests has given a new dimension to the understanding of the disease and enabled the identification of genetically positive probands. However genetic screening is still a matter of research and the variable penetrance of ARVC complicates the clinical significance of a positive finding. During the past 20 years, task force criteria have been useful for diagnosis of ARVC in index patients. However such criteria have demonstrated a low sensitivity in the early stages of the disease. To increase the sensitivity in family screening of patients with proven ARVC, modifications to the Task Force Criteria have recently been proposed [27].

Family screening relies on symptoms and findings in ECG, signal average-ECG, echocardiography and MRI. However echocardiographic abnormalities in the right ventricular wall are still difficult to detect as they can be localised to small areas. Furthermore, visual analysis of RV function from two dimensional grey scale imaging is subjective and can be unreliable for follow-up. New echocardiographic techniques such as measuring the amplitude of tricuspid annular motion (TAPSE) and measurement of myocardial wall velocity using tissue Doppler have improved the accuracy of RV wall motion analysis [28-30]. In recent years assessment of RV myocardial deformation [strain (S) and strain rate (SR) by tissue Doppler has been used in different populations [31]. However, the angle dependency of Doppler S and SR is particularly relevant for the right ventricular free wall and its triangular, oblique shape in the 4 -chamber view. Speckle tracking echocardiography is less dependent on the angle of insonation. Speckle tracking has been well validated in vitro and in vivo. Clinical application of this method to left ventricular function already exists [32-36]. The use of speckle tracking for the assessment of right ventricular function is less investigated but studies are slowly accumulating [37-39].

Using a combination of echocardiographic parameters that were significant in discriminant analysis, we therefore developed an index for the right and the left ventricle based on dimensions and function. Values for these indices in excess of 1 suggest that such an individual expresses features of disturbed ventricular function. While all healthy control subjects had RV and LV indices lower than 1, some of the first- degree relatives deviated from this normal pattern. It is noteworthy that the LV index was frequently abnormal, supporting our previous finding that LV involvement may be an earlier feature of the disease than generally appreciated [21,22]. It should be kept in mind that a substantial number of relatives are non-carriers of the disease gene and their normal echocardiographic findings tend to dilute the effect of reduced measurements on group mean values. An interesting finding in the discriminant analysis was the high impact of myocardial velocity and longitudinal strain based on speckle tracking determined in the right ventricular free wall. Compared to these two measurements, TAPSE was not an independent significant factor. This is in agreement with previous findings that systolic velocity and strain are more sensitive in detecting changes in right ventricular function than TAPSE [40]. Our proposed index, combining different echocardiographic parameters may increase the detection of subclinical changes in relatives.

We suggest that using left and right ventricular indices may be useful, as an abnormal index may be an early sign of the disease. In patients with an established diagnosis of ARVC, the index method may be used as an objective tool for following the progress of the disease*.*

### Limitations

In this study we have focused upon male patients as well as their male relatives and matched controls, to avoid that potential gender differences in myocardial function would influence the relationships studied by adding a smaller number of females to the study group at this stage.

Inclusion of larger number of patients and relatives would strengthen the results, however expanding numbers is difficult due to the rarity of the disease. It is too early to assess the discriminating power of the proposed index based on this cohort of males, since the group is rather small and needs external validation in independent cohorts. The index is intended to continue to be tested as future first degree relatives are identified and entered into the screening programme.

## Conclusion

Measurement of longitudinal strain based on speckle tracking was feasible for RV functional analysis in first degree relatives and in the majority of index patients with ARVC. Strain data of individual LV or RV segments were significantly lower in patients than in controls but did not differ between first degree relatives of ARVC patients and control. We have developed an index incorporating a combination of LV and RV dimensional and functional parameters derived from echocardiography that is proposed for the detection of subclinical echocardiographic abnormalities. In combination with genetic testing, this index might help to find early phenotype expression in mutation carriers relatives of ARVC patients, but needs validation in external and preferably larger populations of first degree relatives of ARVC patients.

## Abbreviations

ARVC: Arrhythmogenic right ventricular cardiomyopathy; ECG: Electrocardiogram; SAECG: Signal average electrocardiogram; LBBB: Left bundle branch block; RBBB: Right bundle branch block; AVO: Aortic valve opening; AVC: Aortic valve closure; LVOT VTI: Left ventricular outflow tract velocity time integral; RVOT/BSA: Right ventricular outflow tract related to body surface area; RVIT/BSA: Right ventricular inflow tract related to body surface area; 2D-: Two dimensional; ROI: Region of interest; Sw: Systolic velocity on tissue Doppler imaging; Ew: Early diastolic velocity on tissue Doppler imaging; Aw: Late diastolic velocity on tissue Doppler imaging; TAPSE: Tricuspid annular plane systolic excursion.

## Competing interests

The authors have no competing interests.

## Authors’ contributions

MÅA planned the study, investigated all patients, performed measurements and analyses and played a major part in the writing of the manuscript. EN took part in planning the study, reviewed and discussed the manuscript text. JE performed measurements and analyses and took part in writing and reviewing the manuscript. LB took part in the statistical analysis and reviewing the manuscript. All authors have read and approved the final manuscript.
